# Supraclavicular Lymphadenopathy: Initial Manifestation of Metastasis in Carcinoma of Cervix

**DOI:** 10.1155/2013/409196

**Published:** 2013-02-26

**Authors:** Mutahir A. Tunio, Mushabbab Al Asiri, Reham Mohamed, Sadeq Al-Dandan

**Affiliations:** ^1^Radiation Oncology, Comprehensive Cancer Center, King Fahad Medical City, Riyadh 59046, Saudi Arabia; ^2^Comprehensive Cancer Center, King Fahad Medical City, Riyadh 59046, Saudi Arabia

## Abstract

*Introduction*. Carcinoma of cervix rarely metastasizes to cervical lymph nodes and is associated with poor prognosis. To date, only few case reports have been reported in the medical literature. Here, we report a case of this unusual manifestation of carcinoma of cervix. *Case Presentation*. A 39-year-old Saudi woman who was treated three years ago for bulky IB stage carcinoma of cervix with total abdominal hysterectomy and bilateral salpingo-oophorectomy and adjuvant chemoradiation presented to us during her routine follow-up visit with left supraclavicular lymphadenopathy. Staging workup revealed additional para-aortic nodal and osseous metastases. The biopsy of left supraclavicular mass confirmed the diagnosis of carcinoma of the cervix. Patient was started on chemotherapy and bisphosphonates. *Conclusion*. Supraclavicular lymph nodes are a rare site of metastasis in carcinoma of cervix, and this can be explained by outlining the drainage of the lymphatic system from the cervix. Supraclavicular lymphadenopathy is associated with variable prognosis.

## 1. Introduction

The incidence of invasive cervical cancer is decreasing in the United States [1]. However, cervical cancer continues to be a major women's health issue in many countries because of inadequate cytological screening programs, and many patients present locally advanced or metastatic FIGO stages IIB-IVB. 

The pattern of metastasis in carcinoma of cervix initially involves pelvic lymph nodes, followed by para-aortic nodes and distant sites. The most frequent metastatic sites are the lungs, extrapelvic nodes, liver, and bones [2]. However, due to multidisciplinary treatment and increased survival, different metastatic sites have been reported, including the cervical lymph nodes. However, supraclavicular lymphadenopathy from carcinoma of cervix is extremely rare. Only few case reports have been published so far in the medical literature [3, 4]. The presence of supraclavicular lymphadenopathy is associated with grave prognosis for survival outcome in patients with carcinoma of cervix.

Here, we present a case report of 39-year-old Saudi woman, who presented with left supraclavicular lymphadenopathy as initial metastatic site after 3 years of treatment for FIGO IB bulky carcinoma of cervix.

## 2. Case Presentation

A 39-year-old Saudi woman presented in our oncology clinic for her routine visit with left lower neck swelling. She had noticed this swelling for 2 months, and it had been rapidly increasing in size over a month causing pain, for which she was taking nonsteroidal anti-inflammatory drugs (NSAIDs), but no benefit. Her previous medical history revealed that three years ago she was treated with total abdominal hysterectomy and bilateral salpingo-oophorectomy (TAH and BSO) followed by adjuvant chemoradiation for bulky FIGO stage IB. She had no history comorbid conditions and no history of smoking, and her weight was stable. On physical examination, her vitals were stable. A fixed, solitary, hard neck mass of size 4 × 4 cm was palpable in the left supraclavicular region. There was no other palpable cervical lymphadenopathy, and the examination of chest, heart, nervous system, abdomen, and pelvis was normal. Clinical differential diagnosis was tuberculosis or carcinoma of breast or lung. 

**Figure 1:**
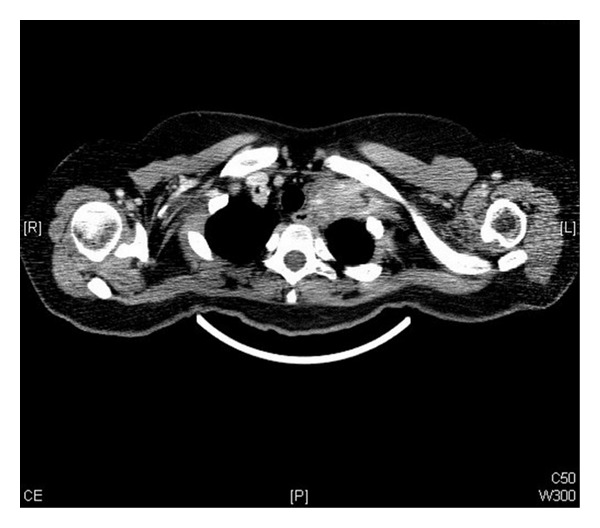
Computed tomography (CT) axial images of neck showing left supraclavicular solid mass of size 3.2 × 2.5 cm encasing the vessels.

**Figure 2:**
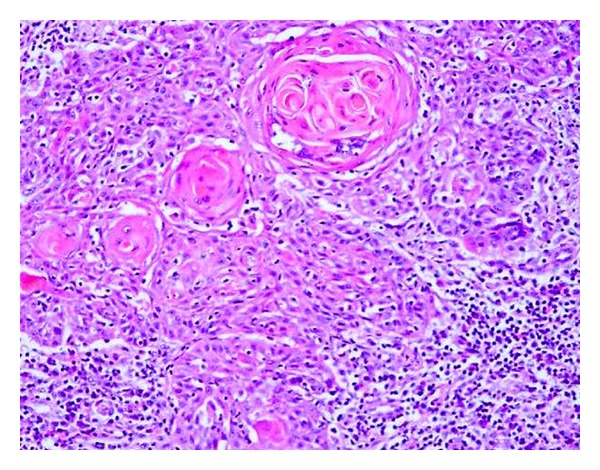
Histopathology of left supraclavicular node showing metastatic squamous cells with abundant eosinophilic cytoplasm and cell keratinization.

**Figure 3:**
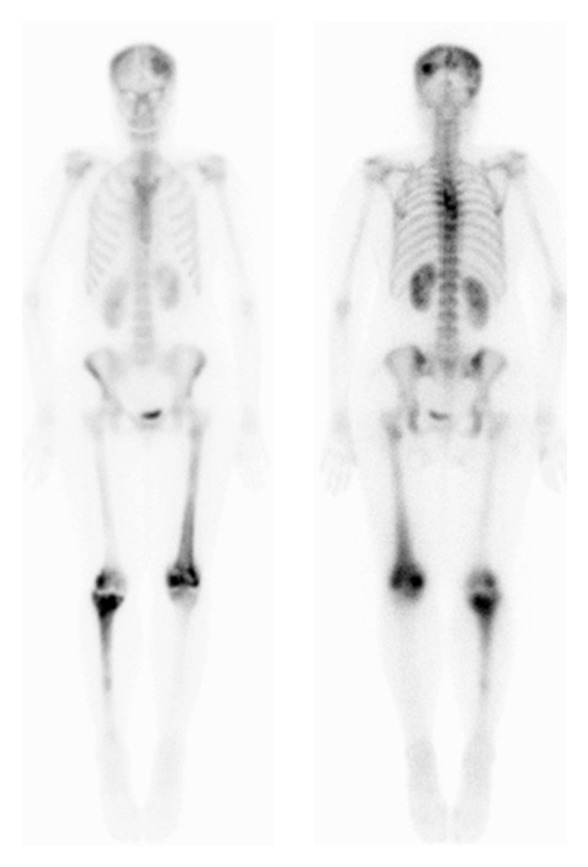
Bone scan showing bone metastasis in skull, dorsal spine, left femur, distal right femur, and proximal right tibia.

Computed tomography (CT) neck showed left supraclavicular solid mass of size 3.2 × 2.5 cm encasing the vessels (Figure 1). The mammogram of both breasts was normal with no solid or cystic lesion. Hematological, renal, and liver function tests, tuberculin, and serum electrolytes were within normal limits. The core biopsy of mass was performed, which revealed metastatic squamous cell carcinoma consisting with cervix primary (Figure 2). CT of chest, abdomen, and pelvis revealed para-aortic lymphadenopathy and lytic lesion in dorsal spine however, no local recurrence. Bone scintigraphy confirmed bone metastasis in skull, dorsal spine, left femur, distal right femur, and proximal right tibia (Figure 3). 

**Figure 4:**
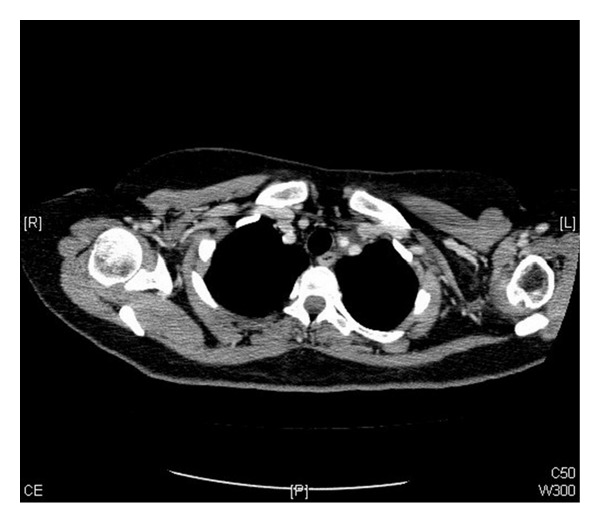
Postchemotherapy CT scan axial images showing a decrease in the size of the left supraclavicular nodes.

Patient was started on duplet chemotherapy (cisplatin and paclitaxel). After four cycles, she has responded well to the treatment with a reported decrease in the size of the supraclavicular nodes (Figure 4).

## 3. Discussion

The reported incidence of metastasis of carcinoma of the cervix to left supraclavicular nodes is 0.1%–1.5% [5]. To date, only few case reports have been published [6, 7]. The manifestation of supraclavicular lymphadenopathy indicates high tumor burden and poor prognosis in patients with carcinoma of cervix. Henriksen [5], in his retrospective review of 18 cases of cervical cancer with supraclavicular lymphadenopathy, has reported a survival time of between 1 and 16 months after the appearance of metastases. 

Possible pattern of spread of tumor cells carcinoma of cervix to the supraclavicular region is best understood through its lymphatic drainage. The carcinoma of the cervix spreads by internal and external iliac lymph nodes from primary lesion, then common iliac and para-aortic lymph nodes, then into the thoracic duct. The thoracic duct communicates with the systemic venous system in the neck at the junction of the left subclavian and internal jugular vein. The left-sided supraclavicular nodes represent the final common path of the body's infradiaphragmatic lymphatic drainage [8]. 

Our patient has responded well to cisplatin and paclitaxel based palliative chemotherapy with partial response and is alive at 6 months after the initial presentation of supraclavicular lymphadenopathy. However, recent trials have documented response rates of 27% achieved by cisplatin in combination with paclitaxel [9, 10]. 

A previous study of 33 cases with cervical cancer and supraclavicular metastasis reported that the SCC-Ag < 15 ng/mL at initial diagnosis and staging/restaging including 18-flouro-deoxyglucose positron emission tomography (FDG-PET) to be associated with a better prognosis [11–13]. However, these tests were not carried out in our patient but are highly recommended for prompt treatment. Further, our patient's TAH+BSO is an inadequate therapeutic model for cervical cancer IB, and there was no complete staging at the time of the first operation, which possibly resulted in cancer recurrence later. 

In conclusion, supraclavicular lymphadenopathy secondary to carcinoma of cervix is rare, and prognosis in such patients is usually poor but not incurable, and treatment is mainly palliative. However, the incorporation of FDG-PET and SCC-Ag markers can be helpful. 

## Abbreviations

CT: Computed tomography
NSAID: Nonsteroidal anti-inflammatory drugs
TAH: Total abdominal hysterectomy
BSO: Bilateral salpingo-oophorectomy
FIGO: International Federation of Gynecologists and Obstetrics
FDG-PET: Flouro-deoxyglucosepositron emission tomography.

## References

[1] Siegel R, Naishadham D, Jemal A (2012). Cancer statistics 2012. *CA: A Cancer Journal for Clinicians*.

[2] Waggoner SE (2003). Cervical cancer. *The Lancet*.

[3] Diddle AW (1972). Carcinoma of the cervix uteri with metastases to the neck. *Cancer*.

[4] Manoharan M, Satyanarayana D, Jeyarajah AR (2008). Cervical lymphadenopathy—an unusual presentation of carcinoma of the cervix: a case report. *Journal of Medical Case Reports*.

[5] Henriksen E (1949). The lymphatic spread of carcinoma of the cervix and of the body of the uterus. A study of 420 necropsies. *American Journal of Obstetrics and Gynecology*.

[6] Shin MS, Shingleton HM, Partridge EE, Nicolson VM, Ho KJ (1995). Squamous cell carcinoma of the uterine cervix: patterns of thoracic metastases. *Investigative Radiology*.

[7] Scott I, Bergin CJ, Muller NL (1986). Mediastinal and hilar lymphadenopathy as the only manifestation of metastatic carcinoma of the cervix. *Canadian Association of Radiologists Journal*.

[8] Ellison E, LaPuerta P, Martin SE (1999). Supraclavicular masses: results of a series of 309 cases biopsied by fine needle aspiration. *Head and Neck*.

[9] Moore DH, Blessing JA, McQuellon RP (2004). Phase III study of cisplatin with or without paclitaxel in stage IVB, recurrent, or persistent squamous cell carcinoma of the cervix: a Gynecologic Oncology Group study. *Journal of Clinical Oncology*.

[10] Kim JY, Kim JY, Kim JH (2012). Curative chemoradiotherapy in patients with stage IVB cervical cancer presenting with paraaortic and left supraclavicular lymph node metastases. *International Journal of Radiation, Oncology, Biology and Physics *.

[11] Qiu JT, Ho KC, Lai CH (2007). Supraclavicular lymph node metastases in cervical cancer. *European Journal of Gynaecological Oncology*.

[12] Ho K-C, Wang C-C, Qiu J-T (2011). Identification of prognostic factors in patients with cervical cancer and supraclavicular lymph node recurrence. *Gynecologic Oncology*.

[13] Tran BN, Grigsby PW, Dehdashti F, Herzog TJ, Siegel BA (2003). Occult supraclavicular lymph node metastasis identified by FDG-PET in patients with carcinoma of the uterine cervix. *Gynecologic Oncology*.

